# Supplementary material for: Strong Negative Association between Cesarean Delivery and Early Initiation of Breastfeeding Practices among Vietnamese Mothers—A Secondary Analysis of the Viet Nam Sustainable Development Goal Indicators on Children and Women Survey
Source: Nutrients. 2023 Oct 24;15(21):4501. doi: 10.3390/nu15214501 (PMC10647442; doi:10.3390/nu15214501)
Supplement: Supplementary file 1 [file nutrients-15-04501-s001.zip › nutrients-2635605-supplementary(1).pdf]

Table S1: Socio-demographic characteristics of mothers who practiced early initiation of breastfeeding in the vaginal-delivery group.

Variables	EIBF# n (%)	No EIBF# n (%)	Total n=1,075(%)	p-value
Age (years)				0.023*
15-17	6 (14.6)	35 (85.4)	41 (3.8)	
18-29	200 (30.6)	454 (69.4)	654 (60.8)	
30-39	124 (35.6)	224 (64.4)	348 (32.4)	
40-49	13 (40.6)	19 (59.4)	32 (3.0)	
Ethnicity of household head				0.743**
Kinh/Chinese	158 (32.4)	329 (67.6)	487 (45.3)	
Others[a]	185 (31.5)	403 (68.5)	588 (54.7)	
Residential regions				0.171*
Northern Midland, Mountain and Central Highlands areas	177 (34.4)	337 (65.6)	514 (47.8)	
Red River and Mekong River Delta areas	76 (27.0)	206 (73.0)	282 (26.2)	
North Central and Central Coastal areas	44 (30.8)	99 (69.2)	143 (13.3)	
Southeast areas	46 (33.8)	90 (66.2)	136 (12.7)	
Living areas				0.294**
Urban	78 (35.0)	145 (65.0)	223 (20.7)	
Rural	265 (31.1)	587 (68.9)	852 (79.3)	
Level of education				0.473*
None/pre-primary	51 (33.3)	102 (66.7)	153 (14.2)	
Primary school	44 (31.7)	95 (68.3)	139 (12.9)	
Junior and senior high school	170 (30.1)	395 (69.9)	565 (52.6)	
Higher education[b]	78 (35.8)	140 (64.2)	218 (20.3)	
Wealth index				0.895*
Poorest/poor	227 (32.0)	482 (68.0)	709 (66.0)	
Middle	47 (33.1)	95 (66.9)	142 (13.2)	
Rich/richest	69 (30.8)	155 (69.2)	224 (20.8)	
Marital status				0.697**
Married/in a union	332 (31.8)	712 (68.2)	1044 (97.1)	
Not in union[c]	11 (35.5)	20 (64.5)	31 (2.9)	
Partner's age[d] (years old)				0.125**
< 31	170 (29.8)	401 (70.2)	571 (54.7)	
≥ 31	162 (34.2)	311 (65.8)	473 (45.3)	
Age at the time of the first delivery (years)				0.076*
< 18	46 (24.9)	139 (75.1)	185 (17.2)	
18-29	280 (33.3)	561 (66.7)	841 (78.2)	
≥ 30	17 (34.7)	32 (65.3)	49 (4.6)	
Parity				0.946*
1	103 (31.2)	227 (68.8)	330 (30.7)	
2-3	202 (32.3)	424 (67.7)	626 (58.2)	
≥4	38 (31.9)	81 (68.1)	119 (11.1)	
Intended pregnancy for the last child				0.132**
Yes	278 (33.1)	563 (66.9)	841 (78.2)	
No	65 (27.8)	169 (72.2)	234 (21.8)	
Total	343 (31.9)	732 (68.1)	1075 (100.0)	

*Chi-square test ** Fisher's exact test

Early initiation of breastfeeding.

[a] Others included minor ethnicities such as Tay, Thai, Muong, Nung, Khmer, and Mong.

[b] Higher education: Women who graduated from vocational school, college, university, or had a postgraduate degree.

[c] Women were single, separated, divorced, or widowed.

[d] Partner's age was divided by the median.

Table S2: Characteristics of antenatal care and delivery of mothers who practiced early initiation of breastfeeding in the vaginal-delivery group.

Variables	EIBF[#] n (%)	No EIBF[#] n (%)	Total n=1,075	p-value
Gestational week at the time of the first ANC[a] of the last pregnancy (weeks)				0.753*
≥13	6 (40.0)	9 (60.0)	15 (1.4)	
1-12	302 (32.0)	643 (68.0)	945 (87.9)	
None	35 (30.4)	80 (69.6)	115 (10.7)	
Number of ANC visits during the last pregnancy (times)				0.513*
1-3	76 (30.9)	170 (69.1)	246 (22.9)	
4-7	106 (30.0)	247 (70.0)	353 (32.8)	
≥ 8	126 (34.9)	235 (65.1)	361 (33.6)	
None	35 (30.4)	80 (69.6)	115 (10.7)	
ANC provider during the last pregnancy				0.030*
Doctor	281 (31.5)	612 (68.5)	893 (83.1)	
Nurse/midwife	14 (30.4)	32 (69.6)	46 (4.3)	
Community health worker	13 (61.9)	8 (38.1)	21 (2.0)	
None	35 (30.4)	80 (69.6)	115 (10.7)	
Place of the delivery of the last child				0.984*
Public hospital	218 (31.6)	471 (68.4)	689 (64.1)	
Other public sectors[b]	43 (33.3)	86 (66.7)	129 (12.0)	
Private medical sectors[c]	15 (32.6)	31 (67.4)	46 (4.3)	
Home/other	67 (31.8)	144 (68.2)	211 (19.6)	
Delivery mode of the last child				
Vaginal delivery	343 (31.9)	732 (68.1)	1075 (100.0)	
Cesarean section	-	-	-	
Delivery assistants for the last child				0.480*
Doctor	252 (31.6)	545 (68.4)	797 (74.1)	
Nurse/midwife only	21 (30.9)	47 (69.1)	68 (6.3)	
Community health worker	8 (50.0)	8 (50.0)	16 (1.5)	
No medical support	62 (32.0)	132 (68.0)	194 (18.0)	
Skin-to-skin contact immediately after the last delivery				0.108**
Yes	243 (33.6)	481 (66.4)	724 (67.3)	
No	100 (28.5)	251 (71.5)	351 (32.7)	
Sex of the last child				0.169**
Male	185 (33.9)	361 (66.1)	546 (50.8)	
Female	158 (29.9)	371 (70.1)	529 (49.2)	
Birth weight of the last child (g)				0.281*
< 2,500	8 (22.2)	28 (77.8)	36 (4.1)	
2,500-3,999	262 (32.2)	552 (67.8)	814 (92.8)	
≥4,000	11 (40.7)	16 (59.3)	27 (3.1)	
Total	343 (31.9)	732 (68.1)	1075 (100.0)	

*Chi-square test ** Fisher's exact test

[#] Early initiation of breastfeeding.

[a] ANC: Antenatal care

[b] Other public sectors referred to local clinics, commune health centers, hospitals of a ministry or sector, and other public institutions.

[c]Private medical sectors were private hospitals or other private medical institutions.

Table S3: Univariate and multivariate analyses of early initiation of breastfeeding among the vaginal-delivery group of Vietnamese mothers.

Variables	OR (CI 95%)	Adjusted-OR (CI 95%)
Age (years)		
15-17	0.39 (0.16-0.94)*	0.14 (0.02-0.81)*
18-29	1 (reference)	1 (reference)
30-39	1.26 (0.95-1.66)	1.11 (0.71-1.75)
40-49	1.55 (0.75-3.21)	1.54 (0.58-4.10)
Ethnicity of household head		
Kinh/Chinese	1 (reference)	1 (reference)
Others[a]	0.96 (0.74-1.24)	0.87 (0.57-1.33)
Residential regions		
Northern Midland, Mountain and Central Highlands areas	1 (reference)	1 (reference)
Red River and Mekong River Delta areas	0.70 (0.51-0.97)*	0.59 (0.38-0.91)*
North Central and Central Coastal areas	1.21 (0.77-1.87)	0.75 (0.45-1.23)
Southeast areas	1.39 (0.89-2.16)	0.67 (0.39-1.17)
Living areas		
Urban	1 (reference)	1 (reference)
Rural	0.84 (0.62-1.15)	0.87 (0.58-1.31)
Level of education		
None/pre-primary	1 (reference)	1 (reference)
Primary school	0.93 (0.57-1.51)	0.70 (0.32-1.54)
Junior and senior high school	0.86 (0.59-1.26)	0.54 (0.26-1.14)
Higher education[b]	1.11 (0.72-1.72)	0.70 (0.30-1.60)
Family wealth index		
Poor/poorest	0.95 (0.65-1.40)	1.04 (0.64-1.70)
Middle	1 (reference)	1 (reference)
Rich/richest	0.90 (0.57-1.41)	0.79 (0.48-1.29)
Partner's age[c] (years old)		
< 31	1 (reference)	1 (reference)
≥ 31	1.23 (0.95-1.60)	1.30 (0.85-1.99)
Age at the time of the first delivery (years)		
< 18	0.66 (0.46-0.95)*	0.81 (0.46-1.41)
18-29	1 (reference)	1 (reference)
≥ 30	1.06 (0.58-1.95)	0.63 (0.30-1.35)
Parity		
1	1 (reference)	1 (reference)
2-3	1.05 (0.79-1.40)	0.69 (0.46-1.02)
≥ 4	1.03 (0.66-1.62)	0.64 (0.28-1.44)
Intended pregnancy for the last child		
No	1 (reference)	1 (reference)
Yes	1.28 (0.93-1.77)	1.21 (0.82-1.80)
Gestational week at the time of the first ANC[d] of the last pregnancy (weeks)		

≥ 13	1.42 (0.50-4.02)	1.47 (0.45-4.77)
1-12	1 (reference)	1 (reference)
None	0.93 (0.61-1.42)	1.47 (0.51-4.26)
Number of ANC visits during the last pregnancy (weeks)		
1-3	0.83 (0.59-1.18)	0.76 (0.43-1.35)
4-7	0.80 (0.59-1.10)	0.80 (0.55-1.16)
≥ 8	1 (reference)	1 (reference)
None	0.82 (0.52-1.28)	-
ANC provider during the last pregnancy		
Doctor	1 (reference)	1 (reference)
Nurse/midwife	0.95 (0.50-1.81)	1.41 (0.62-3.23)
Community health worker	3.54 (1.45-8.64)***	31.16 (2.97-327.52)**
None	0.95 (0.63-1.45)	-
Place of delivery of the last child		
Public hospital	1 (reference)	1 (reference)
Other public sectors[e]	1.08 (0.72-1.61)	1.15 (0.72-1.85)
Private medical sectors[g]	1.05 (0.55-1.98)	0.98 (0.50-1.92)
Home/other	1.01 (0.72-1.40)	0.96 (0.176-5.21)
Delivery assistant for the last child		
Doctor	1 (reference)	1 (reference)
Nurse/midwife only	0.97 (0.57-1.65)	0.97 (0.52-1.81)
Community health worker	2.16 (0.80-5.83)	0.74 (0.90-6.10)
No medical support	1.12 (0.73-1.42)	0.87 (0.28-12.53)
Skin-to-skin contact immediately after the last delivery		
Yes	1 (reference)	1 (reference)
No	0.79 (0.60-1.04)	0.58 (0.37-0.93)*
Sex of the last child		
Male	1 (reference)	1 (reference)
Female	0.83 (0.64-1.08)	0.92 (0.68-1.25)
Birth weight of the last child (g)		
≤2,499	0.60 (0.27-1.34)	0.57 (0.24-1.32)
2,500-3,999	1 (reference)	1 (reference)
≥4,000	1.45 (0.66-3.17)	1.40 (0.61-3.21)

* $p<0.05$, ** $p \leq 0.01$, *** $p \leq 0.001$

[a] Others included minor ethnicities such as Tay, Thai, Muong, Nung, Khmer, and Mong.

[b] Higher education: Women who graduated from senior high school, vocational school, college, university, or had a postgraduate degree.

[c] Partner's age was divided by median.

[d] ANC: Antenatal care

[e] Other public sectors referred to local clinics, commune health centers, hospitals of a ministry or sector, and other public institutions.

[g] Private medical sectors were private hospitals or other private medical institutions.
